# A Review of Hearing Loss Associated with Zika, Ebola, and Lassa Fever

**DOI:** 10.4269/ajtmh.18-0934

**Published:** 2019-07-22

**Authors:** Samuel C. Ficenec, John S. Schieffelin, Susan D. Emmett

**Affiliations:** 1 Tulane University School of Medicine, New Orleans, Louisiana;; 2 Department of Head and Neck Surgery and Communication Sciences, Duke University School of Medicine, Durham, North Carolina;; 3 Duke Global Health Institute, Durham, North Carolina;; 4 Center for Health Policy and Inequalities Research, Duke University, Durham, North Carolina

## Abstract

The neglected tropical diseases Zika, Ebola, and Lassa fever (LF) have all been noted to cause some degree of hearing loss (HL). Hearing loss is a chronic disability that can lead to a variety of detrimental effects, including speech and language delays in children, decreased economic productivity in adults, and accelerated cognitive decline in older adults. The objective of this review is to summarize what is known regarding HL secondary to these viruses. Literature for this review was gathered using the PubMed database. Articles were excluded if there were no data of the respective viruses, postinfectious complications, or conditions related to survivorship. A total of 50 articles were included in this review. Fourteen articles discussing Zika virus and subsequent complications were included. Across these studies, 56 (21.2%) of 264 Zika-infected individuals were found to have HL. Twenty-one articles discussing Ebola virus and subsequent complications were included, with 190 (5.7%) of 3,350 Ebola survivors found to have HL. Fifteen additional articles discussing LF and subsequent complications were included. Of 926 individuals with LF, 79 (8.5%) were found to have HL. These results demonstrate a relationship between HL and infection. The true prevalence is likely underestimated, however, because of lack of standardization of reporting and measurement. Future studies of viral sequelae would benefit from including audiometric evaluation. This information is critical to understanding pathophysiology, preventing future cases of this disability, and improving quality of life after survival of infection.

## INTRODUCTION

Tropical diseases have immense societal impact due in large part to their myriad long-term sequelae. Classically, these disabilities include physical impairments such as blindness, limb and physical deformities, an increased number of negative maternal and neonatal outcomes, and delayed physical or mental development.^1,2^ Furthermore, the association of these illnesses with poverty and the loss of productivity resulting from these disabilities lead to increased levels of stigma and social isolation, which contributes to the total burden of disease.^3–6^ The calculation and comparison of the number of disability-adjusted life years (DALYs) lost because of neglected tropical diseases (56.6 million DALYs) to other more common diseases such as HIV/AIDS (84.5 million DALYs) and malaria (46.5 DALYs) illustrates the large impact of these diseases on the populations they affect.^1,4,5^ Hearing loss (HL) is an often neglected and understudied sequelae of these infections, which contributes to the number of DALYs lost. Hearing loss affects more than 1.3 billion people worldwide and is now the 4th leading cause of years lived with disability.^7^ The effects of HL are lifelong and span from speech and language delays in childhood to restricted employment opportunities in adults and accelerated cognitive decline in older adults.^8–14^ The global burden of HL is unequally distributed, with more than 80% of affected individuals living in low- and middle-income countries, the very places where access to hearing care is limited.

Viruses were first established as an etiology of HL in the 1950s and are suspected to contribute to 12.8–25% of sudden-onset HL cases.^15–17^ Zika, Ebola, and Lassa fever (LF) are all tropical diseases which have received little worldwide attention until recent epidemics, and each of these viruses has been reported to be associated with HL. By comparing the prevalence reported for these and other viruses, Zika, Ebola, and LF may be associated with HL prevalence, that is, up to 300× greater than that of more common and better understood viral etiologies.^18–21^ The true burden of HL secondary to Zika, Ebola, and LF is unknown, however, and may be underreported because of lack of proper measurement of this chronic disability. Despite the paucity of data, the World Health Organization (WHO) recognizes the potential public health impact of these associations and has requested a review of the existing literature on Zika, Ebola, and LF for the upcoming World Report on Hearing, to be released in 2020. The objective of this review is, therefore, to describe what is known regarding HL secondary to these three tropical diseases, identify gaps in knowledge, and propose areas of research to increase our understanding of pathophysiology and potentially lead to new treatment modalities for viral-mediated HL.

## METHODS

This literature search and analysis was conducted from August 2018 through April 2019. All study designs, publication dates, and languages were considered. Literature was gathered from PubMed using key terms and Boolean operators. Key terms used included the following: Zika, Ebola, Lassa, Survivors, Sequelae, HL, Hearing Impairment, Deafness, Complications, Congenital, and Post-Ebola Syndrome. Abstracts and titles of all retrieved studies were reviewed for mention of secondary complications, and the full texts of relevant articles were obtained. Articles were excluded if there were no data or discussion of the respective viruses, postinfectious complications, or conditions related to survival but not directly caused by the virus itself. Data regarding demographics and HL were gathered and aggregated according to the respective cause of infection. Preferred Reporting Items for Systematic Reviews and Meta-Analyses (PRISMA) guidelines were followed as applicable in creation of this review.

## RESULTS

**Figure 1.**
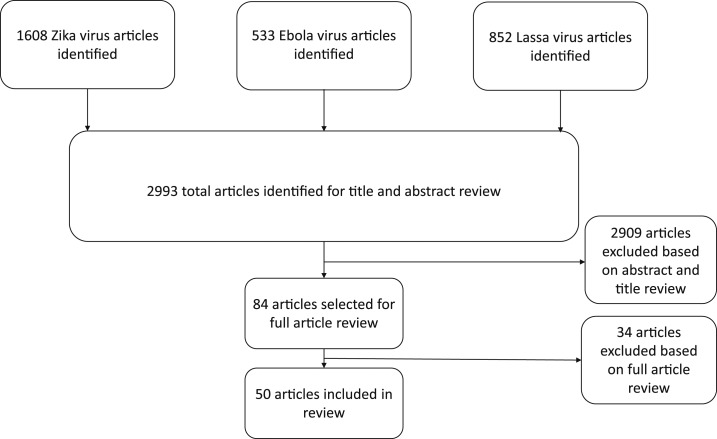
Literature search results.

Two thousand nine hundred ninety-three total articles were identified by this methodology. Of these, 2,909 articles were excluded based on abstract and title review and 34 articles were excluded based on full article review. A total of 50 articles were included in the analysis (Figure 1).

### Hearing loss and Zika.

Fourteen articles discussing Zika virus and subsequent complications in 347 individuals were included in this review (Table 1). Across studies, 56 (21.2%) of 264 individuals were found to have some degree of HL.^18,22–34^ Four of the fourteen articles described acquired HL in adults following Zika infection (Table 1). The HL in these cases varied from moderate to severe and was reported as both unilateral and bilateral, with most patients experiencing recovery to normal or previous thresholds.^22–24,30^ Ten articles presented complication data of congenital Zika syndrome related to HL (Table 1).^18,22,25–29,33–35^ The majority of these studies used standard HL screening methods for infants, including measurement of auditory brainstem response and otoacoustic emission, which assesses cochlear function.^18,25–29,31,33,34^ The proportion of infants with reported HL in these studies varied from 6% to 68%. One article presented in-depth testing of two individuals, one of whom had moderate unilateral HL and one with normal hearing thresholds. Importantly, the patient with HL was also found to have poor speech recognition scores in the same ear. These studies suggest the association of Zika virus not only with HL but also with auditory processing disorders such as auditory neuropathy. The wide range of HL prevalence found by this analysis indicates the need for further research on this disability.

**Table 1:** Adult and congenital Zika hearing loss (HL) findings by year

First author	Publication year	Study type	Sample size (*n*)	Age group	HL Screening Method	HL result (*n*, %)	Unilateral HL (*n*)	Bilateral HL (*n*)	Control group (*n*, % HL)
Tappe^23^	2014	Case report	1	Adult	Self-report	1 (100)	NR	NR	ND
M.E.R.G^29^	2015	Cross-sectional	23*	Neonatal	OAE	2 (9)	NR	NR	ND
Leal^18^	2016	Retrospective cohort	70	Pediatric	ABR to click and tone burst stimuli	5 (6)	NR	NR	ND
Leal^25^	2016	Case series	2	Neonatal	Transient OAE followed by ABR to click stimuli	1 (50)	NR	NR	ND
Vinhaes^24^	2017	Case series	3	Adult	Audiometry	3 (100)	1	2	ND
Martins^22^	2017	Case series	2	Adult	Audiometry	1 (50)	1	0	ND
Satterfeldt^27^	2017	Cross-sectional	19	Pediatric	Physician-reported HINE assessment	13 (68)	NR	NR	ND
Santos^28^	2017	Case series	2	Neonatal	Evoked OAE followed by ABR	1 (50)	NR	NR	ND
Wheeler^26^	2018	Cross-sectional	47	Pediatric	No response to voice or sound	13 (28)	NR	NR	ND
					Does not look for sound	8 (17)			
					No response to word “No”	20 (43)			
de Laval^30^	2018	Prospective cohort	49	Adult	NR	NR	NR	NR	ND
Ventura^31^	2018	Case report	1	Neonatal	ABR to click stimuli	1 (100)	1	0	ND
Franca^32^	2018	Cross-sectional	8	Pediatric	NR	NR	NR	NR	ND
Vianna^33^	2019	Prospective cohort	26	Pediatric	ABR	2 (8)	NR	NR	65 (3)
Calle-Giraldo^34^	2019	Prospective cohort	68	Neonatal	ABR	6 (9)	3	3	ND

ABR = auditory brainstem response; ND = not done; NR = not reported; OAE = otoacoustic emission; HINE = Hammersmith infant neurological examination Adult, 18 years or greater; pediatric, 0–24 months; neonatal, anomalies detected at birth.

* Total sample size of 104, only 23 screened for HL.

### Hearing loss and Ebola.

Twenty-one articles discussing Ebola virus and subsequent complications in 5,055 individuals were included in this review (Table 2). Of the 3,385 individuals studied, 223 (6.6%) were found to have some degree of HL using audiometric evaluation and survey instruments.^19,36–55^ Only one of 21 articles used audiometry to objectively measure HL. This study, by Rowe et al.,^19^ recruited convalescent Ebola survivors and household contacts following the conclusion of the 1995 Ebola outbreak in Kikwit, DRC. The study defined HL as an inability to hear at least 1 frequency between 0.5 and 4 kHz at 25 dB. The authors reported that 18 (64.3%) of 27 individuals developed HL after surviving Ebola infection, and 11 of these patients had developed HL within the first 6 months following discharge from Ebola treatment centers. At the end of the 21-month follow-up period, seven individuals (26%) were found to have persistent HL. The remainder of the articles that described HL as sequelae of Ebola relied on questionnaires or self-report of symptoms.^36–46,50,52–54^ The proportion of individuals reporting HL in these articles varied widely, from 0% to 22%. In these studies relying on self-report, HL typically arose late in the course of the disease and persisted throughout recovery. Self-reported timing of HL onset was broad, spanning from the initial hospital admission to as many as 350 days post discharge.^36–40,42^ Despite continued complaints of HL, the lone study to measure HL by audiometry demonstrated resolution in several individuals.^19^ Thus, it is possible that HL related to Ebola may resolve spontaneously. Data regarding Ebola-related HL is scarce, and more studies are required to elucidate the persistence of this disability.

**Table 2:** Ebola hearing loss (HL) findings by hearing screening method by year

First author	Publication year	Study type	Sample size (*n*)	Median age (years)	HL screening method	HL results (*n*, %)	Days to HL onset (median DPI)	Control group (*n*)
Rowe^19^	1999	Prospective cohort	29	27	Audiometry	18 (64.3)	< 180	152 (NR)
Bwaka^42^	1999	Retrospective cohort	103	38	Self-Reported	13 (12.6)	NR	ND
Clark^43^	2015	Retrospective cohort	70	40	Questionnaire	13 (27)	NR	223 (10)
Qureshi^38^	2015	Cross-sectional	105	38.9*	Questionnaire	0 (0)	NR	ND
Mattia^37^	2016	Cross-sectional	277	29	Self-report	17 (6)	14	ND
Jacobs^36^	2016	Case report	1	39	Self-report	1 (100)	11	ND
Tiffany^39^	2016	Prospective cohort	166	24.7†	Self-reported	5 (3)	31–60	ND
Nanyonga^46^	2016	Cross-sectional	81	29	Questionnaire	NR	NR	ND
Fallah^49^	2016	Retrospective cohort	70	NR	NR	NR	NR	ND
Etard^40^	2017	Cross-sectional	802	28.4	Self-reported	19 (2.4)	350	ND
Shantha^41^	2017	Cross-sectional	96	38.6	Self-reported	10 (10.4)	NR	ND
Hereth-Hebert^48^	2017	Prospective cohort	341	26	NR	NR	NR	ND
Wilson^45^	2018	Cross-sectional	242	30	Questionnaire	4 (1.6)	NR	ND
Jagadesh^44^	2018	Retrospective case control	27	NR	Questionnaire	5 (18.5)	NR	54
Kelly^47^	2018	Cross-sectional	20	53.2*	NR	NR	NR	187 (NR)
Wing^50^	2018	Retrospective cohort	137	25	Self-report	30 (22)	NR	ND
Overholt^51^	2018	Prospective cohort	299	31	NR	NR	NR	ND
Howlett^52^	2018	Case series	35	28	Self-report	3 (8.6%)	NR	ND
de St. Maurice^53^	2018	Cross-sectional	329	33†	Questionnaire	19 (6)	NR	ND
Kelly^55^	2019	Prospective cohort	859	12–50+†	NR	NR	NR	ND
PREVAIL^54^	2019	Prospective cohort	966	NR	Self-report	66 (6.8%)	NR	2,350 (2.2)

DPI = days postinfection; ND = not done; NR = not reported.

* Age reported as mean age of sample.

† Only range of ages reported.

### Hearing loss and LF.

Fifteen articles discussing LF and subsequent complications in 1,207 individuals were included in this review (Table 3).^20,56–69^ Of 15 articles, 11 presented HL data.^20,56–65^ Across studies, 53 (6.0%) of 898 individuals were found to have some degree of HL using audiometric evaluation and survey instruments.^20,56–64^ Thirty-eight (71.7%) affected individuals were found to have bilateral HL, and 15 (28.3%) individuals demonstrated unilateral HL. Audiometry was used to characterize HL in five of 11 studies.^20,56–69^ The mean pure-tone average (PTA) for all reported data was 66.5 dB, which is consistent with severe HL. This measurement was gathered from 139 (15.5%) of 898 individuals with an average age of 33.7 years. Several studies monitored progression of HL. Eleven of 22 individuals were found to have residual HL at 1 year, and one reported residual loss 4 years after the initial infection.^20,57–59,62,63^ At the end of the 1 year period, nine of these individuals were found to have severe HL, including three cases of bilateral HL and six cases of unilateral.^20^ Cummins and colleagues included three separate evaluations to characterize the HL secondary to LF infection.^20^ In the third evaluation, a case-control study of 32 individuals with HL in comparison with 32 individuals without, 26 (81.2%) of 32 individuals with HL were found to be seropositive for LF antibodies versus only six (18.7%) of those without HL. Interestingly, only 13 (50%) seropositive individuals with HL were aware that LF might be the cause of HL.^20^ These studies indicate that LF may be an underappreciated cause of HL in LF endemic areas. This analysis finds that the prevalence of LF-related HL ranges widely, from 0% to 81.25%. More robust studies are needed to determine the relationship between symptomatic disease, HL and seropositivity.

**Table 3:** Lassa Fever HL Findings by year

First author	Publication year	Study type	Sample size	Mean age (years)	HL screening method	HL results (*n*, %)	Average severity of HL*	Unilateral HL	Bilateral HL	Days to HL onset (median DPI)	Control group (*n*, % HL)
White^63^	1972	Case series	23	26.6	Self-report	4 (17.4)	NR	NR	NR	NR	ND
Mertens^64^	1973	Cross-sectional	10	20–56‡	Self-report	3 (30%)	NR	NR	NR	NR	ND
Grundy^57^	1980	Case report	1	25	Self-report	1 (100)	NR	1	0	14	ND
McCormick^60^	1987	Case–control	430	NR	NR	12 (2.8)	NR	3	9	10–15	ND
Frame^68^	1987	Cross-sectional	33	< 1†	NR	NR	NR	NR	NR	NR	ND
Hirabayashi^69^	1988	Case report	1	48	NR	NR	NR	NR	NR	NR	ND
Frame^67^	1989	Retrospective	246	NR	NR	NR	NR	NR	NR	NR	ND
Cummins^20^	1990	Prospective cohort	49	30.2	Audiometry	14 (28.6)	Severe	7	14	5-12	ND
Cummins^20^	1990	Case–control	51	30.3	Audiometry	9 (17.6)	Moderate	3	6	NR	45 (4)
Günther^66^	2001	Case report	1	56	NR	NR	NR	NR	NR	NR	ND
Macher^61^	2006	Case series	2	34.5	Audiometry	1 (50)	NR	1	0	NR	ND
Okokhere^56^	2009	Case series	2	31	Audiometry	2 (100)	Severe	0	2	9	ND
Ibekwe^62^	2011	Prospective cohort	37	35.3	Audiometry	5 (13.5)	Severe	0	5	NR	37 (0)
Grahn^59^	2016	Case report	1	72	Self-report	1 (100)	NR	0	1	22	ND
Choi^58^	2018	Case report	1	46	Self-report	1 (100)	NR	0	1	5	ND
Okokhere^65^	2018	Retrospective cohort	291	35	NR	0 (0)	NR	NR	NR	NR	ND

DPI = days postinfection; HL = hearing loss; ND = not done; NR = not reported.

* Severity determined based on WHO standards.

† Ordinal data presented were used to calculate median age.

‡ Only range of ages reported.

## DISCUSSION

The major lifelong sequelae of neglected tropical diseases are secondary disabilities following infection. HL is an understudied morbidity following these viral tropical disease pathogens. This review examines the association between HL and Zika, Ebola, and LF viruses, summarizing what is known regarding this complication. Results of this analysis demonstrate the range of HL prevalence associated with each of these viruses. The major limitation is the small amount of data that exist to fully characterize this disability and the lack of studies that follow any standard protocol of measurement or reporting, which likely underestimates the true prevalence and undermines data quality. Furthermore, few studies attempt repeat screening to detect progression or late-onset HL. Social stigma and psychiatric complaints are reported complications of both HL and these tropical diseases.^8,9,70–74^ However, no studies have examined the relationship between these complications.

Important similarities and differences were observed in HL associated with the three viruses. Zika virus, a well-described cause of birth defects, may also lead to various levels of HL in both acquired and congenital cases. Interestingly, in-depth audiology testing in the acquired cases of Zika-related HL suggested the presence of a sound processing disorder called auditory neuropathy.^75,76^ The association of Zika with this type of retrocochlear HL has important public health implications because auditory neuropathy is not identified with typical methods of hearing screening. Hearing loss in congenital Zika cases was commonly reported in children older than 1 year, suggesting that Zika may lead to delayed onset of HL and emphasizing the need for recurring screening beyond the newborn period.^21^ The most well-supported hypothesis of the mechanism for Zika-related HL is direct viral invasion of neurons in the auditory pathway.^77–79^ The virus has been shown to have preference for undifferentiated neurons, which may explain the devastating complications resulting from congenital cases in comparison with adults and may serve as a possible explanation for the observed auditory neuropathy findings.^28,80^

In contrast to the auditory neuropathy observed in Zika, HL associated with Ebola and LF is similar to common viral-mediated etiologies of HL and can be easily detected with traditional hearing screening. Prevalence of Ebola- and LF-mediated HL is likely underestimated in this review because of the paucity of studies using objective audiometric measurements. The one study that used objective audiometric testing found a staggering 64.3% of Ebola survivors developed some form of postinfectious HL.^19^ However, because of the low quality of data presented by these studies, it must be emphasized that these results must be interpreted with caution, highlighting the urgent need for future studies of HL secondary to tropical diseases. Various mechanisms have been proposed for the etiology of post-Ebola syndrome symptoms, depicted by detection of the virus in areas such as the eye and semen, including the persistence of virus in immune-privileged sites leading to a direct cytopathic effect.^37,40,81–84^ It has been theorized that the penetration of the virus into cerebrospinal fluid may allow passage into the perilymph via the cochlear aqueduct, leading to the development of Ebola-associated HL. Although most cases of LF are thought to be asymptomatic or subclinical, sequelae, including HL, have been noted to occur across all severities of disease.^20,62,85–87^ The pathogenesis responsible for LF-related HL is poorly understood, and both immunologic and cytopathic mechanisms have been proposed.^88,89^

### Future directions.

Hearing loss is a growing public health concern with lifelong impact. As DALYs and the economic impact secondary to HL continue to increase, it is imperative that steps are taken to address this disability. These results demonstrate that the prevalence of HL associated with Zika, Ebola, and LF infection may be up to 300× greater than common viral etiologies of HL. It is critical that future studies of these tropical infections include objective audiological evaluation using a standard, WHO-supported definition of HL, coupled with longitudinal rescreening to accurately determine the prevalence and fully characterize the natural course of HL secondary to these viruses. Furthermore, future studies of sequelae can provide evidence of causal relationships between these tropical viruses and HL, identify risk factors for diagnosis and prognostication, and elucidate mechanisms leading to HL. Such knowledge is crucial to the development of public health interventions to prevent this understudied disability and improve the quality of life after survival from these devastating and neglected tropical diseases.
